# Diagnostic Value of Serum and Salivary Podoplanin as Clinical Biomarkers for Distinguishing Oral Cancer from Oral Leukoplakia

**DOI:** 10.3390/diagnostics16020206

**Published:** 2026-01-09

**Authors:** Hafize Uzun, Guven Bozarslan, Seyma Dumur, Naile Fevziye Misirlioglu, Mehmet Nuri Elgormus, Canan Duvarcı, Remise Gelisgen, Aysegul Batioglu Karaaltin, Yetkin Zeki Yilmaz

**Affiliations:** 1 Department of Biochemistry, Faculty of Medicine, Istanbul Atlas University, 34303 Istanbul, Turkey; seyma_dumur@hotmail.com (S.D.); nailemisirlioglu@gmail.com (N.F.M.); 2 Faculty of Dentistry, Istanbul Atlas University, 34303 Istanbul, Turkey; guven.bozarslan@st.atlas.edu.tr; 3 Department of Otolaryngology Head and Neck Surgery, Faculty of Medicine, Istanbul Atlas University, 34303 Istanbul, Turkey; mehmetnuri.elgormus@atlas.edu.tr; 4 Department of Biochemistry, Cerrahpasa Medical School, Istanbul University-Cerrahpasa, 34098 Istanbul, Turkey; canan.duvarci@iuc.edu.tr (C.D.); remise.gelisgen@iuc.edu.tr (R.G.); 5 Department of Otolaryngology Head and Neck Surgery, Cerrahpasa Medical Faculty, Istanbul University-Cerrahpasa, 34098 Istanbul, Turkey; a.batioglukaraaltin@iuc.edu.tr (A.B.K.); yetkin.yilmaz@iuc.edu.tr (Y.Z.Y.)

**Keywords:** podoplanin, oral cancer, oral leukoplakia, biomarker, salivary diagnostics, ROC analysis

## Abstract

**Objective:** This study aimed to evaluate serum and salivary podoplanin (PDPN) levels in patients with oral cancer (OC) and oral leukoplakia (OL) and to investigate their potential role as diagnostic biomarkers in distinguishing between these conditions. **Materials and Method:** Ninety participants were enrolled: 30 healthy controls, 30 patients with OL, and 30 patients with histopathologically confirmed OC. All cases were recruited from the Department of Otorhinolaryngology, Cerrahpaşa Medical Faculty and Istanbul Atlas University Hospital. Demographic characteristics, comorbidities, and biochemical parameters were recorded. Serum and salivary PDPN levels were measured using the ELISA method. **Results:** Serum PDPN levels were significantly higher in the OC group (3.25 ± 0.80 ng/mL) compared with both OL (1.85 ± 0.56 ng/mL) and controls (0.98 ± 0.42 ng/mL) (*p* < 0.001). Salivary PDPN levels showed a similar pattern, being highest in OC (2.65 ± 0.75 ng/mL), followed by leukoplakia (1.40 ± 0.45 ng/mL), and controls (0.72 ± 0.30 ng/mL) (*p* < 0.001). Importantly, both serum and salivary PDPN concentrations increased progressively with increasing epithelial dysplasia severity among patients with OL (one-way ANOVA, *p* < 0.001). ROC analysis demonstrated excellent diagnostic accuracy for OC: AUC = 0.976 for serum PDPN (cut-off: 2.0 ng/mL; sensitivity 93.3%, specificity 100%) and AUC = 0.987 for salivary PDPN (cut-off 1.24 ng/mL; sensitivity 93.3%, specificity 95%). **Conclusions:** Serum and salivary PDPN levels were significantly elevated in patients with OC and demonstrated excellent diagnostic performance in distinguishing malignant lesions from OL and healthy controls. The observed stepwise increase in PDPN levels with dysplasia severity further supports its role in malignant transformation. Notably, salivary PDPN represents a non-invasive, practical, and reproducible biomarker that may aid in early detection and risk stratification of high-risk oral premalignant lesions. PDPN assessment could therefore complement clinical and histopathological evaluation, although larger prospective studies are warranted to validate its diagnostic and prognostic utility.

## 1. Introduction

Oral cancer (OC) represents a significant global health burden and is influenced by lifestyle-related factors. According to recent GLOBOCAN data, lip and oral cavity cancer accounted for approximately 389,000 new cases worldwide in 2022 [1]. OCs are primarily classified by histopathological origin, with oral squamous cell carcinoma (OSCC) comprising the majority of cases, followed by less common malignancies such as salivary gland tumors, lymphomas, melanomas, and sarcomas. This classification underpins diagnostic accuracy, prognostic assessment, and the identification of biomarkers and therapeutic targets, thereby supporting personalized patient management [2]. Risk factors, which include drinking alcohol, smoking, human papillomavirus (HPV) infection, a pro-inflammatory factor-rich diet, and poor oral hygiene, must be known and avoided by the general population [3]. Intraoral examination methods such as inspection and palpation in the detection of oral mucosal lesions have an important place in early diagnosis and reduce mortality. There are several diagnostic techniques, which can be divided into currently available procedures and new developing technologies. Adjunctive diagnostic tools have been increasingly incorporated into clinical practice [4]. In addition to conventional examination, techniques such as vital staining, chemiluminescence-based devices (e.g., ViziLite^®^, MicroLux™ DM), and autofluorescence imaging systems (e.g., VELscope^®^, OralID^®^) are used to enhance lesion visualization and guide biopsy site selection for the confirmation of OSCC [5]. However, the variable specificity and operator dependency of these methods highlight the need for objective, biomarker-based diagnostic approaches.

Oral leukoplakia (OL) is the most common oral potentially malignant disorder (OPMD) and carries a variable risk of malignant transformation, which is increased in non-homogeneous lesions, large lesion size, lateral tongue involvement, smoking, and the presence of epithelial dysplasia [6]. Since its initial definition by the World Health Organization (WHO) in 1978, diagnostic criteria for OL have been periodically refined to improve clinical identification [7].

Podoplanin (PDPN) is a type I transmembrane glycoprotein involved in platelet aggregation and lymphatic development [8,9,10]. Beyond its physiological roles, PDPN has emerged as a promising diagnostic biomarker and potential therapeutic target in OSCC [11]. Through interactions with proteins such as CLEC-2, CD44, galectin-8, CCL21, and cytoskeletal adaptor molecules, PDPN regulates signaling pathways that promote tumor cell migration, invasion, and survival, and elevated PDPN expression has been associated with enhanced tumor growth and reduced apoptotic activity [12,13].

OSCC, the predominant form of OC, is strongly associated with modifiable lifestyle factors, particularly tobacco use and alcohol consumption, which synergistically contribute to oral carcinogenesis by inducing chronic inflammation and molecular alterations. These processes may influence the expression of tumor-associated biomarkers, including PDPN. Although previous studies have shown that PDPN tissue expression increases with higher grades of epithelial dysplasia [14,15], existing evidence is largely limited to immunohistochemical analyses and prognostic evaluation.

While tissue PDPN expression correlates with the severity of epithelial dysplasia [16,17], current research remains largely restricted to immunohistochemical prognostications. Consequently, the diagnostic utility of circulating and salivary PDPN particularly their capacity to differentiate OSCC from premalignant lesions is poorly defined and lacks simultaneous comparative evaluation. This study addresses this gap by systematically assessing serum and salivary PDPN levels in patients with OSCC and OL, as well as in healthy individuals. Using multivariate and ROC analyses, we evaluated the non-invasive potential of PDPN as a clinical tool for the early detection and differential diagnosis of oral malignancies.

## 2. Materials and Methods

### 2.1. Ethical Approval

This study received approval from the Ethics Committee of Istanbul Atlas University Medical Faculty (protocol number: E-22686390-050.99-25527, Date: 30 March 2023) and informed consent was obtained from all subjects involved in the study. The study was conducted in compliance with the Declaration of Helsinki.

### 2.2. Study Design and Setting

This study was designed as a prospective, case–control investigation conducted at the Department of Otorhinolaryngology, Cerrahpaşa Medical Faculty and Department of Otorhinolaryngology, Istanbul Atlas University Hospital. Patient recruitment and sample collection were conducted prospectively between April 2023 and December 2024 at the participating centers. The methodological framework was informed by current literature on salivary and serum biomarker profiling in potentially malignant oral disorders and OSCC [18,19]. Epithelial dysplasia was graded as mild, moderate, or severe according to the WHO classification criteria based on histopathological evaluation of biopsy specimens. High-grade dysplasia is defined as moderate or severe. The design, data-collection tools, and analytical strategies were developed to ensure that the selected methods were fully compatible with the study objectives namely, to compare serum and salivary biomarker levels between patients with dysplastic or malignant oral lesions and healthy controls, and to evaluate their diagnostic potential.

The methodology encompasses research design, sampling procedures, dependent and independent variables, biological sample processing, and statistical analyses. All study procedures were structured and mapped to corresponding work packages in the project plan.

### 2.3. Participants and Diagnostic Criteria

**Figure 1:**
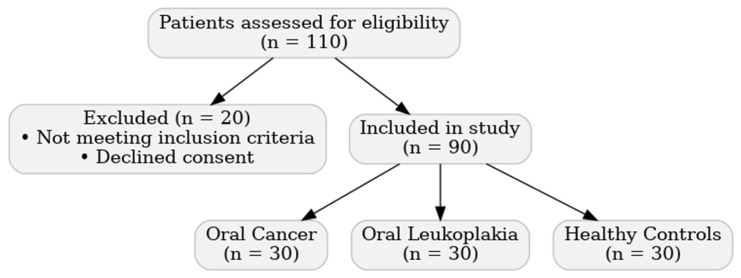
Flowchart of patient recruitment, exclusion process, and final group allocation.

A total of 90 participants were enrolled in this prospective study (Figure 1).

Inclusion Criteria

*Case group:*

(i) Patients presenting with clinically suspicious oral lesions who subsequently received a definitive diagnosis of primary OC or OL based on histopathological examination; (ii) For the OL group, only lesions with histologically confirmed epithelial dysplasia were included, ensuring a homogeneous premalignant cohort; (iii) Age ≥ 18 years; (iv) No history of chemotherapy or radiotherapy prior to sample collection.

*Exclusion Criteria*

(i) Presence of any malignancy other than primary OC in potential case participants; (ii) History of chemotherapy or radiotherapy; (iii) Any systemic disease or relevant medical history in control participants; (iv) Previous diagnosis of cancer in another system; (v) Regarding medication use, participants’ current and recent drug histories were reviewed during enrollment. Individuals receiving treatments known to significantly affect inflammatory or oncologic biomarkers (e.g., immunosuppressive therapy, systemic corticosteroids, or chemotherapy) were excluded: (vi) Declining or inability to sign informed consent.

*Control group:*

(i) Volunteers attending the same dental outpatient clinic with no oral mucosal pathology and no systemic disease; (ii) Age ≥ 18 years; (iii) Ability to read, understand, and sign the informed consent document.

### 2.4. Venous Blood Sample Collection and Processing

Following informed consent, a single venous blood sample (minimum 5 mL) was collected from each participant into a yellow-cap, gel-separator vacuum tube. All phlebotomies were performed by experienced medical personnel, and participants were monitored for potential adverse events. Samples were centrifuged at 3000× *g* for 10 min, after which serum aliquots were transferred into polypropylene cryovials. Until biochemical analysis, all serum samples were stored at −80 °C to prevent degradation of target biomarkers, consistent with best-practice recommendations for biobanking and biomarker research.

### 2.5. Saliva Sample Collection and Processing

Saliva sampling was conducted according to validated unstimulated whole saliva collection protocols frequently used in OC biomarker studies (15). Participants received the following standardized instructions: No food, beverage, or smoking for at least 1 h prior to sampling. Seated position with the head slightly tilted forward. Avoid swallowing saliva and refrain from speaking during collection. Collection of unstimulated whole saliva using the drooling/spitting technique into a sterile, graded funnel-attached container. Each participant was instructed to continuously expectorate for 8–10 min, yielding a sample reflecting secretions from major and minor salivary glands as well as gingival crevicular fluid. Samples were centrifuged at 2500× *g* for 10 min. The resulting supernatant was aliquoted into sterile cryovials and stored at −80 °C until biochemical assays.

### 2.6. Measurement of Serum and Salivary PDPN Levels

Serum and salivary PDPN concentrations were measured using a quantitative sandwich enzyme-linked immunosorbent assay (ELISA) method. Measurements were performed in duplicate according to the manufacturer’s instructions using the Human Podoplanin (PDPN) ELISA Kit (Cat. No: E-EL-H1810; Elabscience^®^, Wuhan, China). The minimum detectable dose of Human PDPN is 0.094 ng/mL (the sensitivity of this assay, or lowest detectable limit was defined as the lowest protein concentration that could be differentiated from zero). Coefficients of variation were <10%. Absorbance was read at 450 nm using a microplate reader (BioTek^®^ ELx800, ELx 800 UV; BioTek Instruments, Winooski, VT, USA), and concentrations were calculated from the standard calibration curve generated by four-parameter logistic regression.

### 2.7. Statistical Analysis

Statistical analyses were performed using the SPSS 26.0 software package (SPSS Inc., Chicago, IL, USA). The sample size was calculated using the G*Power software (version 3.1), assuming a medium effect size and achieving a statistical power greater than 80%. Data analyses included ROC curve analysis, multiple linear regression, logistic regression, and Pearson correlation. Group comparisons were conducted using *t*-tests or ANOVA where appropriate. A *p*-value < 0.05 was considered statistically significant.

Statistical analyses were performed using the SPSS 26.0 software package (SPSS Inc., Chicago, IL, USA). The sample size was calculated using G*Power, assuming a medium effect size and achieving a statistical power greater than 80%. Data analyses included receiver operating characteristic (ROC) curve analysis, multiple linear regression, logistic regression, and Pearson correlation analysis. Comparisons between study groups were conducted using independent-samples *t*-tests or one-way ANOVA, as appropriate. Within the OL group, one-way ANOVA was additionally used to compare serum and salivary podoplanin levels across epithelial dysplasia grades (mild, moderate, severe). Furthermore, ROC analyses were extended to assess the ability of serum and salivary podoplanin to discriminate high-grade dysplasia (moderate–severe) from mild dysplasia. A *p*-value < 0.05 was considered statistically significant.

## 3. Results

**Figure 2:**
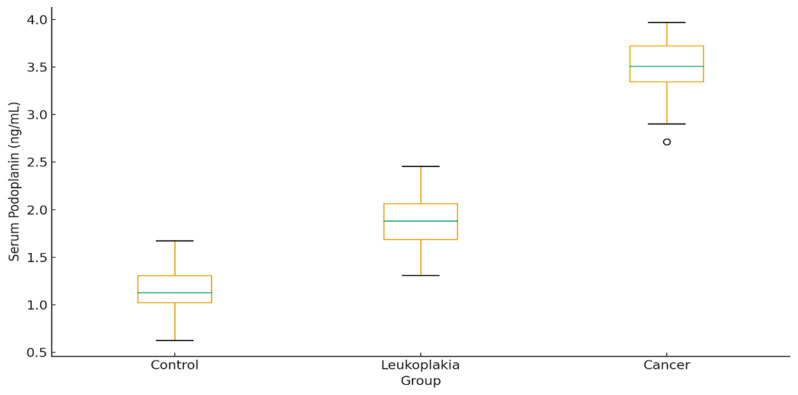
Serum podoplanin levels by group.

**Figure 3:**
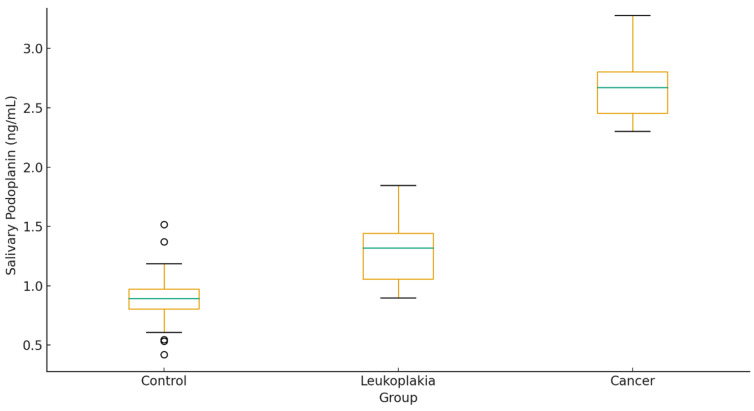
Salivary podoplanin levels by group.

The study included 90 participants: 30 healthy controls, 30 patients with OL, and 30 patients with histopathologically confirmed OC. The mean age was significantly higher in the OC group compared with controls and leukoplakia patients (*p* < 0.001). Male predominance was observed in the cancer group (22/8). Smoking and alcohol use were more common among patients with OC (73% and 40%, respectively). Hypertension and diabetes were the most frequent comorbidities, with significantly higher prevalence in the OC group (*p* = 0.02). Table 1 summarizes clinical and biochemical characteristics with pairwise comparisons. Age, smoking, alcohol use, CRP, glucose, ALT, AST, and both serum and salivary PDPN levels increased progressively from control → leukoplakia → cancer (all *p* < 0.05). PDPN levels were highest in the cancer group: Serum: 3.5 ± 0.9 ng/mL, Saliva: 2.6 ± 0.7 ng/mL (Figure 2 and Figure 3).

Among patients with OL, epithelial dysplasia was classified as mild in 40.0%, moderate in 33.3%, and severe in 26.7% of cases according to WHO criteria (Table 2).

Both serum and salivary PDPN concentrations increased progressively with increasing dysplasia severity in patients with OL (one-way ANOVA, *p* < 0.001) (Table 3).

High-grade dysplasia was defined as moderate or severe epithelial dysplasia in patients with OL. Both serum and salivary PDPN demonstrated strong discriminatory performance for identifying high-risk lesions (Table 4).

**Figure 4:**
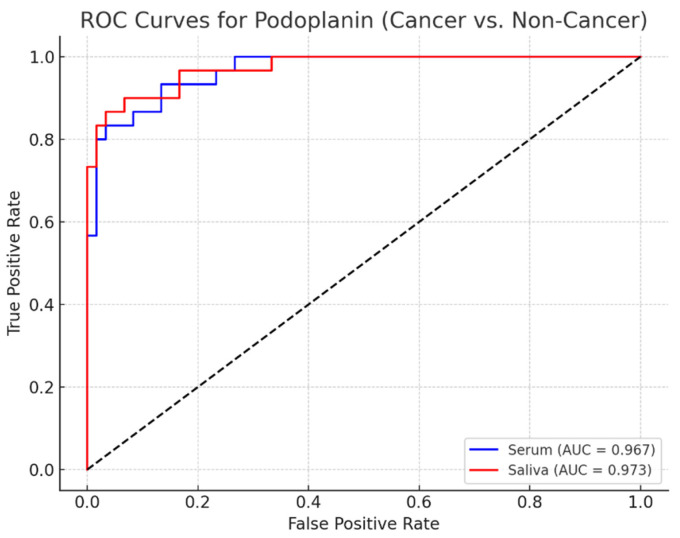
ROC curves for serum and salivary podoplanin.

Serum and salivary PDPN showed excellent diagnostic performance (Table 5). Serum PDPN: AUC = 0.976, Sensitivity 93.3%, Specificity 100%. Salivary PDPN: AUC = 0.987, Sensitivity 93.3%, Specificity 95%. Figure 4 illustrates ROC curves.

OC diagnosis was the strongest predictor of PDPN elevation in both serum and saliva (Table 6). CRP was an independent secondary predictor, supporting an inflammatory component in disease progression.

Both serum and salivary PDPN were strong independent predictors of OC: Serum PDPN: OR = 3.25 (1.9–5.1). Salivary PDPN: OR = 2.95 (1.7–4.6) (*p* < 0.001 for both). CRP showed a modest association (*p* = 0.03) (Table 7).

Serum and salivary PDPN showed a strong positive correlation (r = 0.654).

PDPN levels were highest in patients with combined tobacco and alcohol exposure, suggesting a potential additive or synergistic effect (Table 8).

Smoking and alcohol use demonstrated moderate positive correlations with PDPN levels (Table 9).

## 4. Discussion

The most significant findings of this study demonstrate that both serum and salivary PDPN levels increase progressively from healthy controls to leukoplakia and reach their highest values in patients with OC, indicating a strong association with disease severity. ROC analysis revealed excellent diagnostic accuracy for both biomarkers, with AUC values exceeding 0.97, positioning PDPN as a highly reliable discriminator between malignant and non-malignant lesions. Multiple linear regression identified OC diagnosis and systemic inflammation (CRP levels) as independent contributors to elevated PDPN concentrations, confirming the biomarker’s mechanistic relevance in carcinogenesis. Logistic regression further established serum and salivary PDPN as robust, independent predictors of OC, even after adjustment for confounding factors such as age, smoking, and alcohol use. Additionally, PDPN levels showed substantial correlations with lifestyle-related risk factors, supporting its sensitivity to carcinogenic exposures. Collectively, these findings emphasize PDPN’s diagnostic value and its potential utility as a noninvasive biomarker, particularly in saliva for early detection and clinical differentiation of OC.

Salivary biomarkers are measurable biological molecules present in saliva that reflect physiological and pathological processes within the oral cavity. Owing to its direct contact with oral tissues, saliva contains proteins, enzymes, cytokines, and nucleic acids that are involved in local immune responses and tissue homeostasis. Alterations in salivary biomarker profiles have been associated with common oral diseases, including periodontitis and dental caries, as well as OCs, where they may reflect inflammation, microbial dysbiosis, tissue destruction, and malignant transformation. Consequently, salivary biomarkers offer a non-invasive and clinically accessible means for disease detection, monitoring, and risk assessment in oral health [20]. The present study demonstrates that both serum and salivary PDPN levels are significantly elevated in OC compared with OL and healthy controls, with excellent diagnostic accuracy. Importantly, PDPN levels increased progressively with the severity of epithelial dysplasia, supporting its role in oral carcinogenesis and malignant transformation. These findings extend previous tissue-based observations by demonstrating that PDPN can be reliably detected in biofluids, particularly saliva, offering a non-invasive diagnostic alternative. The strong performance of salivary PDPN highlights its potential utility as a low-cost, repeatable, and non-invasive biopsy marker for early detection and risk stratification of oral premalignant lesions.

PDPN promotes tumor cell migration and invasion by regulating cytoskeletal remodeling and Rho GTPase-dependent signaling pathways, and through its interaction with CLEC-2 contributes to tumor–stromal crosstalk and metastatic potential [9,10,14,15,18,19]. PDPN is frequently expressed in OL. Together with histology, PDPN may serve as a powerful biomarker to predict the risk for OC development in patients with OL [21]. Overexpression of PDPN has been shown to be associated with aggressive tumor behavior and poor clinical outcomes in OC, supporting its role as a prognostic biomarker [22]. PDPN expressions are regulated by several tumor-promoting pathways, including 12-O-tetradecanoylphorbol-13-acetate (TPA), rat sarcoma viral oncogene homolog (RAS), and the proto-oncogene tyrosine-protein kinase Src (SRC). SRC enhances PDPN transcription through the focal adhesion adaptor protein Crk-associated substrate/Breast Cancer Anti-Estrogen Resistance Protein 1 (Cas/BCAR1), which promotes cytoskeletal reorganization and increases tumor cell motility. As a non-receptor tyrosine kinase, SRC supports anchorage-independent growth and migration processes central to invasion and metastatic progression. Although SRC mutations are uncommon, dysregulated SRC activation is well-documented in multiple malignancies, including colorectal, breast, pancreatic, brain, and cutaneous cancers [11,23,24]. The results of our study are highly consistent with these mechanistic pathways. We demonstrated a progressive elevation of serum and salivary PDPN levels from controls to leukoplakia and overt OC, paralleling the shift from non-dysplastic to dysplastic and invasive phenotypes described in SRC- and RAS-driven oncogenic signaling. Moreover, the strong correlations we observed between PDPN levels and clinical risk factors particularly smoking, alcohol use, and systemic inflammation (CRP) further support the concept that environmental carcinogens and inflammatory microenvironmental cues can activate upstream pathways that converge on PDPN upregulation. The excellent diagnostic performance of PDPN in ROC analyses (AUC > 0.97) reinforces its role not merely as a downstream marker but as a functional mediator of tumor progression. Together, the mechanistic insights from the literature and our clinical findings underscore PDPN’s central role in oral carcinogenesis and highlight its value as a biomarker reflecting both molecular pathway activation and disease severity.

Heterogeneous OL has the potential to transform into cancer, with a prevalence ranging from 0.13% to 17.5% or higher in certain regions. Most OSCC cases are not diagnosed in the early stages; therefore, prompt intervention for OL is a crucial preventive strategy for malignant transformation [25]. Nearly 90% of all OCs are squamous cell carcinoma, and major risk factors are chronic irritation, smoking, and alcohol consumption. Nearly 90% of all OCs are squamous cell carcinoma, and major risk factors are chronic irritation, smoking, and alcohol consumption [26]. Most OCs have a premalignant lesion stage [27]. At present, there is no clear consensus regarding whether early aggressive surgical intervention or careful surveillance yields superior clinical outcomes. As a result, there remains a critical unmet need for molecular and biological investigations aimed at improving risk assessment and management strategies for OL. Lip and oral cavity cancers collectively contribute to a considerable proportion of global cancer morbidity and mortality, with region-specific incidence rates highest in South-Central Asia and other areas with prevalent tobacco use [1]. These statistics highlight the significant variation in disease distribution and underscore the importance of improved early detection strategies targeted at high-risk populations. In the present study, the moderate positive correlations observed between PDPN levels and both smoking and alcohol consumption, the potential synergistic effect of combined tobacco and alcohol exposure warrants consideration. Although smoking and alcohol individually showed significant associations with PDPN expression, their combined impact may further amplify inflammatory and oncogenic signaling pathways involved in oral carcinogenesis. Future studies with larger cohorts are needed to specifically evaluate the joint effect of tobacco–alcohol exposure on PDPN regulation.

Evidence indicates that PDPN expression is activated at an early stage of OSCC development and may serve as a marker for premalignant lesions with a high likelihood of malignant progression. In pooled analyses encompassing more than 300 premalignant oral lesions from multiple retrospective studies [21,28,29,30], elevated PDPN expression was consistently associated with an increased risk of transformation to OC.

Recent evidence by He et al. [31] demonstrated that PDPN overexpression is associated with poor prognosis in glioma, highlighting its role as a marker of aggressive tumor behavior. Consistent with these findings, our study demonstrated that both serum and salivary PDPN levels were markedly elevated in patients with OC compared with those with leukoplakia and healthy controls. The strong diagnostic performance and independent predictive value observed in our cohort further support PDPN as a biomarker linked to tumor progression and adverse biological features.

Heguedusch et al. [14] reported that PDPN expression is prominently upregulated in OSCC and is closely associated with invasive tumor fronts, supporting its role in promoting cell motility and facilitating epithelial–mesenchymal transition-like behavior. This aligns strongly with the present study, in which both serum and salivary PDPN levels increased progressively from control to leukoplakia to OC, and were identified as independent predictors of malignancy. Together, these findings reinforce PDPN’s function as a key molecular driver of OC invasiveness and highlight its clinical value as a minimally invasive biomarker for distinguishing malignant from premalignant lesions.

Retzbach et al. [9] demonstrated that PDPN is not only a reliable diagnostic marker but also a functionally significant driver of OC progression, influencing tumor cell migration, invasion, and metastatic potential. PDPN has emerged as a clear chemotherapeutic target for OC. Consequently, compounds that target PDPN are being developed as anticancer reagents. These include antibodies, cell-based immunotherapies, and chemical biologics. These mechanistic insights align closely with our findings, in which both serum and salivary PDPN levels showed a clear stepwise increase from controls to leukoplakia to OC, and exhibited excellent diagnostic performance in ROC analyses. The strong independent predictive value of PDPN in our logistic regression models supports its relevance as a clinically actionable biomarker. Taken together, the functional role proposed by Retzbach et al. [9] and the robust clinical associations observed in our study reinforce PDPN’s utility as both a diagnostic indicator and a potential therapeutic target in OSCC. PDPN has gained increasing recognition as a clinically meaningful biomarker and potential therapeutic target in OC. Its extracellular localization makes PDPN particularly suitable for selective drug delivery strategies aimed at minimizing systemic toxicity. Emerging PDPN-targeted agents hold promises for enhancing early detection, improving therapeutic specificity, and reducing off-target effects. As these technologies advance, they may substantially strengthen diagnostic and treatment options for patients with OC [9].

Identifying oral potentially malignant disorders that carry a high risk of progressing to OC remains a major clinical challenge. Although lesions exhibiting dysplastic features are generally considered at greater risk, it is well established that some OCs arise from lesions without any histologically detectable dysplasia. Moreover, the assessment of epithelial dysplasia is affected by several subjective factors, including tissue quality, sampling site, and both inter- and intra-observer variability [32,33,34,35]. These limitations underscore the fact that the histological evaluation of dysplasia, its presence and severity does not always provide a reliable indicator of malignant transformation risk. Therefore, there is a critical need for additional objective biomarkers capable of improving risk stratification and guiding appropriate clinical management of high-risk lesions.

In addition to oncologic processes, PDPN expression may be influenced by systemic metabolic and inflammatory conditions. Recent evidence has shown that serum PDPN levels are elevated in diabetic nephropathy and correlate with disease progression, suggesting an association between PDPN and chronic metabolic–inflammatory states [36]. PDPN can be considered a novel marker for coronary atherosclerosis. Low-serum PDPN concentrations characterize patients with coronary artery disease [37]. Consistent with these observations, diabetes mellitus and hypertension were more prevalent among patients with OC in the present study. However, multivariate regression analyses demonstrated that neither diabetes mellitus nor hypertension independently predicted serum or salivary PDPN levels, whereas OC status and CRP remained significant. These findings indicate that although metabolic and cardiovascular comorbidities may modulate PDPN levels indirectly, PDPN elevation in OC is predominantly driven by tumor-related inflammatory and oncogenic mechanisms, supporting the robustness of PDPN as a diagnostic biomarker independent of common comorbidities.

This study has several limitations. First, the sample size was relatively small and derived from a single center, which may limit the generalizability of the findings. Second, the cross-sectional design prevents assessment of causal or temporal associations between PDPN levels and disease progression. Third, although major confounders such as age, smoking, alcohol use, and inflammation were adjusted for, unmeasured biological variables may still have influenced PDPN expression. Finally, the study did not include longitudinal follow-up or survival outcomes, preventing evaluation of PDPN as a prognostic marker.

### Clinical Implications and Future Directions

From a clinical perspective, salivary PDPN represents a promising non-invasive, cost-effective, and easily repeatable biomarker with potential applications in the early detection and risk stratification of oral premalignant and malignant lesions. Its ability to reflect both dysplasia severity and malignant transformation suggests a role in screening high-risk populations and in longitudinal monitoring of patients with OL. Future prospective studies with larger, multicenter cohorts are warranted to validate these findings, define standardized diagnostic cut-off values, and explore the integration of PDPN measurement into routine clinical workflows, potentially in combination with existing adjunctive diagnostic modalities.

## 5. Conclusions

This study demonstrates that both serum and salivary podoplanin (PDPN) levels are significantly elevated in patients with OC compared with those with OL and healthy controls, with excellent discriminatory performance in ROC analyses. Multivariate and logistic regression analyses confirmed OC status as an independent determinant of PDPN elevation, even after adjustment for demographic, inflammatory, and lifestyle-related confounders. Importantly, both serum and salivary PDPN concentrations increased progressively with increasing epithelial dysplasia severity in patients with OL, supporting the association between PDPN upregulation and malignant transformation risk. The strong concordance between serum and salivary measurements further reinforces the biological validity of PDPN as a biomarker in oral carcinogenesis.

## Figures and Tables

**Table 1:** Demographic and biochemical characteristics of study groups with pairwise comparisons.

Variable	Control (*n* = 30)	Oral Leukoplakia (*n* = 30)	Oral Cancer (*n* = 30)	p^1^	p^2^	p^3^
**Age** **(years, mean ± SD)**	47.3 ± 10.5	52.8 ± 11.2	58.6 ± 12.1	0.04	<0.001	0.03
**Sex (M/F)**	15/15	18/12	22/8	0.62	0.01	0.27
**Smoking (%)**	27%	47%	73%	0.08	<0.001	0.02
**Alcohol use (%)**	10%	20%	40%	0.21	0.004	0.04
**Hypertension (%)**	13%	20%	27%	0.34	0.01	0.11
**Diabetes Mellitus (%)**	7%	13%	23%	0.42	0.03	0.18
**CRP** **(mg/L, mean ± SD)**	2.1 ± 1.0	3.8 ± 1.6	6.5 ± 2.2	0.01	<0.001	0.01
**Glucose** **(mg/dL, mean ± SD)**	92 ± 12	105 ± 15	118 ± 18	0.02	<0.001	0.04
**Creatinine** **(mg/dL, mean ± SD)**	0.84 ± 0.2	0.96 ± 0.3	1.12 ± 0.4	0.20	0.01	0.09
**ALT** **(U/L, mean ± SD)**	22 ± 6	28 ± 7	35 ± 9	0.03	0.001	0.05
**AST** **(U/L, mean ± SD)**	24 ± 7	30 ± 8	39 ± 10	0.05	0.001	0.04
**Serum podoplanin (ng/mL, mean ± SD)**	1.2 ± 0.4	1.8 ± 0.6	3.5 ± 0.9	0.02	<0.001	0.001
**Salivary podoplanin (ng/mL, mean ± SD)**	0.9 ± 0.3	1.3 ± 0.5	2.6 ± 0.7	0.03	<0.001	0.002

**Abbreviations**: **CRP**, C-reactive protein; **ALT**, Alanine aminotransferase; **AST**, Aspartate aminotransferase. p^1^: Control vs. Leukoplakia; p^2^: Control vs. OC; p^3^: Leukoplakia vs. OC.

**Table 2:** Distribution of epithelial dysplasia grades in the oral leukoplakia group.

Degree of Epithelial Dysplasia	Number of Patients (*n* = 30)	Percentage (%)
***Mild dysplasia***	12	40.0
***Moderate dysplasia***	10	33.3
***Severe dysplasia***	8	26.7

**Table 3:** Serum and salivary PDPN levels according to dysplasia grade.

Dysplasia Grade	Serum PDPN (ng/mL)	Salivary PDPN (ng/mL)	*p*-Value
**Mild dysplasia**	1.4 ± 0.4	1.0 ± 0.3	<0.001
**Moderate dysplasia**	1.9 ± 0.5	1.4 ± 0.4	
**Severe dysplasia**	2.4 ± 0.6	1.9 ± 0.5	

**Table 4:** ROC analysis for high-grade dysplasia (moderate–severe) in oral leukoplakia.

Biomarker	AUC (95% CI)	Cut-Off	Sensitivity (%)	Specificity (%)
**Serum PDPN**	0.91 (0.82–0.98)	1.9	88.0	85.0
**Salivary PDPN**	0.89 (0.79–0.96)	1.3	85.0	83.0

**Table 5:** ROC analysis results for serum and salivary podoplanin.

Biomarker	AUC (95% CI)	Cut-Off	Sensitivity (%)	Specificity (%)
**Serum podoplanin** **(ng/mL)**	0.976 (0.95–0.99)	2.0	93.3	100
**Salivary podoplanin** **(ng/mL)**	0.987 (0.96–0.99)	1.24	93.3	95.0

**Table 6:** Multiple linear regression predicting podoplanin levels.

Dependent Variable	Predictors	β (95% CI)	*p*
**Serum podoplanin (ng/mL)**	OC group (vs. control)	+1.85 (1.1–2.5)	<0.001
	CRP (mg/L)	+0.08 (0.03–0.13)	0.004
**Salivary podoplanin (ng/mL)**	OC group (vs. control)	+1.60 (0.9–2.3)	<0.001
	CRP (mg/L)	+0.05 (0.01–0.09)	0.009

**Table 7:** Logistic regression predicting oral cancer (yes/no).

Variable	OR (95% CI)	*p*-Value
**Serum podoplanin (ng/mL)**	3.25 (1.9–5.1)	<0.001
**Salivary podoplanin (ng/mL)**	2.95 (1.7–4.6)	<0.001
**Age (years)**	1.02 (0.98–1.06)	0.28
**CRP (mg/L)**	1.05 (1.01–1.11)	0.03

**Table 8:** Combined effect of smoking and alcohol use on PDPN levels.

Exposure Group	Serum PDPN (ng/mL)	Salivary PDPN (ng/mL)
**Non-smoker/Non-alcohol**	1.3 ± 0.4	1.0 ± 0.3
**Smoking only**	1.9 ± 0.6	1.4 ± 0.4
**Alcohol only**	1.7 ± 0.5	1.3 ± 0.4
**Smoking + Alcohol**	2.6 ± 0.7	2.1 ± 0.6

**Table 9:** Correlation matrix for podoplanin and clinical variables.

Variable	Serum Podoplanin	Saliva Podoplanin	Smoking	Alcohol	CRP
**Serum_Podoplanin**	1.0	0.654	0.723	0.479	0.01
**Saliva_Podoplanin**	0.654	1.0	0.687	0.441	−0.011
**Smoking**	0.723	0.687	1.0	0.017	−0.047
**Alcohol**	0.479	0.441	0.017	1.0	0.072
**CRP**	0.01	−0.011	−0.047	0.072	1.0

## Data Availability

The raw data supporting the conclusions of this article will be made available by the authors on request.
